# Prevalence of Trichomonas vaginalis infection among Egyptian women using culture and Latex agglutination: cross-sectional study

**DOI:** 10.1186/s12905-015-0169-2

**Published:** 2015-02-07

**Authors:** Ahmed Mahmoud, Nadine A Sherif, Rana Abdella, Amira R El-Genedy, Abdalla Y El Kateb, Ahmed NH Askalani

**Affiliations:** Department of Obstetrics and Gynecology, Faculty of Medicine, Cairo University, Cairo, Egypt; Department of Parasitology, Faculty of Medicine, Cairo University, Cairo, Egypt

**Keywords:** Trichomonas vaginalis, Wet mount examination, Culture, Latex test

## Abstract

**Background:**

This is a cross-sectional study carried out in the Obstetrics and Gynecology Department at Kasr Al- Ainy Cairo University Hospitals.

**Methods:**

One thousand female patients in the child bearing period (age 18-45 yrs) were included in this study. These females were non-pregnant and non-menstruating with no douching or intercourse for at least 2–3 days, no use of antibiotics, anti-protozoal or steroids for the past 15 days complaining of vaginal discharge with or without itching, burning sensation or both. Vaginal swabs were obtained from all patients for examination by direct wet mount examination, Giemsa staining, Modified Diamond culture and latex agglutination test Kalon) to detect the presence of *Trichomonas vaginalis* infection.

**Results:**

The prevalence of trichomonas infection was 50 cases, latex agglutination test detected 50 positive cases, 30 of which were also positive by culture, and only 10 were detected both by Giemsa staining and by wet mount.

The wet mount, Giemsa staining and Kalon latex test had sensitivities of 33.3, 33.3% and 100% respectively while their specificities were 100%, 100% and 97.9% respectively.

**Conclusion:**

Screening tests should be done routinely to depict cases of *T. vaginalis* infection and should be included in the control programs of sexually transmitted infections. Although wet mount is not a sensitive method for diagnosis of *T. vaginalis* yet, it is a good positive one. Staining is only useful when there is heavy *T. vaginalis* infection.

Latex agglutination is a highly sensitive, simple, rapid and cost effective test. It provides results within 2-3 minutes and it has the potential for use in screening and diagnosis of *T. vaginalis* infection.

## Background

Trichomonas vaginalis, is an anaerobic flagellated protozoan parasite, that infects the human urogenital tract [1]. It is the most common curable sexually transmitted infection [2]. According to the World Health Organization (WHO) estimates, the global incidence of new trichomoniasis cases were approximately 250 millions, 24.5 million of these cases being in Europe, making it more prevalent than gonorrhea and chlamydia , however, 7 million occur in the United States [3].

While T. vaginalis is associated with sexual risk behavior and other sexually transmitted infections (STIs), it is now considered an important dependent pathogen. Multiple studies have linked T. vaginalis infection to significant and costly adverse health outcomes, such as pelvic inflammatory disease; vaginitis, cervicitis, urethritis and prostatitis in those also infected with HIV [4].

It was discovered that T. vaginalis infection plays a role in causing ectopic pregnancy, tubal factor of infertility and adverse pregnancy outcomes [5]. Conventional methods for diagnosing T. vaginalis are microscopic examination of wet-mount preparations and culture-based systems. Both methods rely on the collection of viable organisms and suffer from poor sensitivity. The use of sensitive assays for detection of T. vaginalis in a clinical laboratory setting -compared to traditional methods- are clearly needed to improve the detection of T. vaginalis infected cases, especially with the increasing evidence that T. vaginalis may play a significant role in the transmission of human immunodeficiency virus (HIV) [6]. This has been the rationale behind our study where we found the need to determine its prevalence among Egyptian females and produce recommendations concerning a largely accurate and cost-effective screening test to be implemented in the current Egyptian STDs control programs.

### Aim of work

This study aimed at determining the prevalence of T. vaginalis infection in Egyptian women attending the Obstetrics and Gynecology Clinic in Kasr Al-Ainy Hospitals, using conventional diagnostic techniques as wet mount, stain, culture and Latex agglutination test for detection. We have previously studied other causes of vaginitis in our center and limited this study to a large scale cross-sectional study to assess the incidence of Trichomonas.

## Methods

One thousand female patients attending the Gynecology and Obstetrics outpatient clinic of Kasr El -Aini hospitals from December 2011 to August 2013 were included in this cross-sectional study. Prior to enrollment, an informed consent was obtained from each patient. This study was approved by the Science and Ethical Committee of the Obstetrics and Gynecology department (in Sixth OBGYN Department Council for year 2011, ie: June 2011) as well as the General Ethical committee in Kasr Al- Ainy Hospitals (for the same month and year).

We approached women in their childbearing period (18-45years) who complained of vaginal discharge of any type, with itching, burning sensation or both, and with any other gynecological manifestations suggestive of trichomoniasis such as pruritis vulvae, dyspareunia or dysuria. All women were non-pregnant and non-menstruating with no douching or intercourse for at least 2–3 days, no use of antibiotics, anti-protozoal or steroids for the past 15 days.

Each patient was subjected to thorough history taking, PV examination and non-lubricated sterile speculum examination. Then three vaginal swabs were collected from the posterior fornix of the vagina by a sterile cotton wool swab sticks with wooden shafts to be examined as follows:

1. The first swab was kept in tube containing 3 ml sterile Phosphate Buffered Saline (PBS), pH: 7.2, for wet mount microscopy and Giemsa staining.
2. The second swab was kept in one of the Kalon TV latex tubes included with the kits containing sterile PBS, pH 7.2, to perform the latex agglutination.
3. The third swab was inoculated immediately into the culture tube containing modified Diamond’s medium.

Each sample was labeled using a serial number and was quickly transported to the Parasitology laboratory of Kasr Al-Aini Hospitals for complete testing and incubation through a collaborative approach.

**Figure 1:**
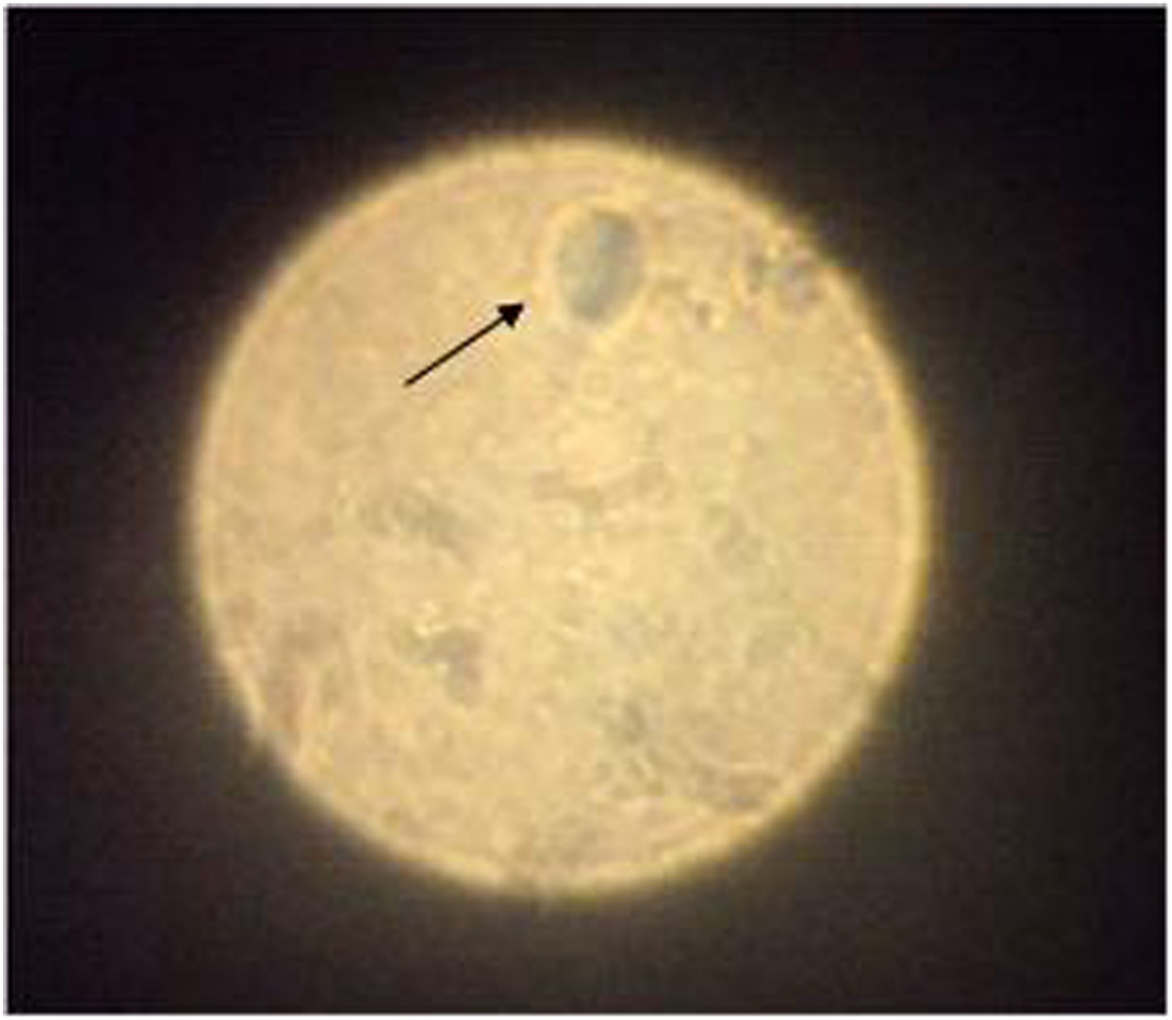
***T. vaginalis*** **in fresh wet mount examination × 40.** The wet preparation of the vaginal discharge was made immediately, by applying a drop from the sample to a small area of a clean glass slide with a cover slip in order not to trap air bubbles. The whole smear was examined using conventional light microscopy for motile *Trichomonas vaginalis* with low power objective (× 10), then with high power objective (× 40). If motile flagellates with characteristic motility (jerky movement) and morphology of trichomonads were seen, the specimen was reported as positive for T. vaginalis. If no flagellated organisms were seen, the specimen was reported as negative for T. vaginalis. The test is dependent on demonstrating the motility of *T. vaginalis* that loses their motility rapidly after leaving the vaginal environment. Wet mount examination was performed within less than half an hour of collection in order to get optimal results and was held at room temperature. The T. vaginalis trophozoite is the oval as well as flagellated, or “pear” shaped as seen on a wet-mount.

The wet preparation from the vaginal discharge was made immediately using a clean glass slide with cover and examined microscopically for motile T. vaginalis under × 10 and × 40 objectives (Figure 1).

**Figure 2:**
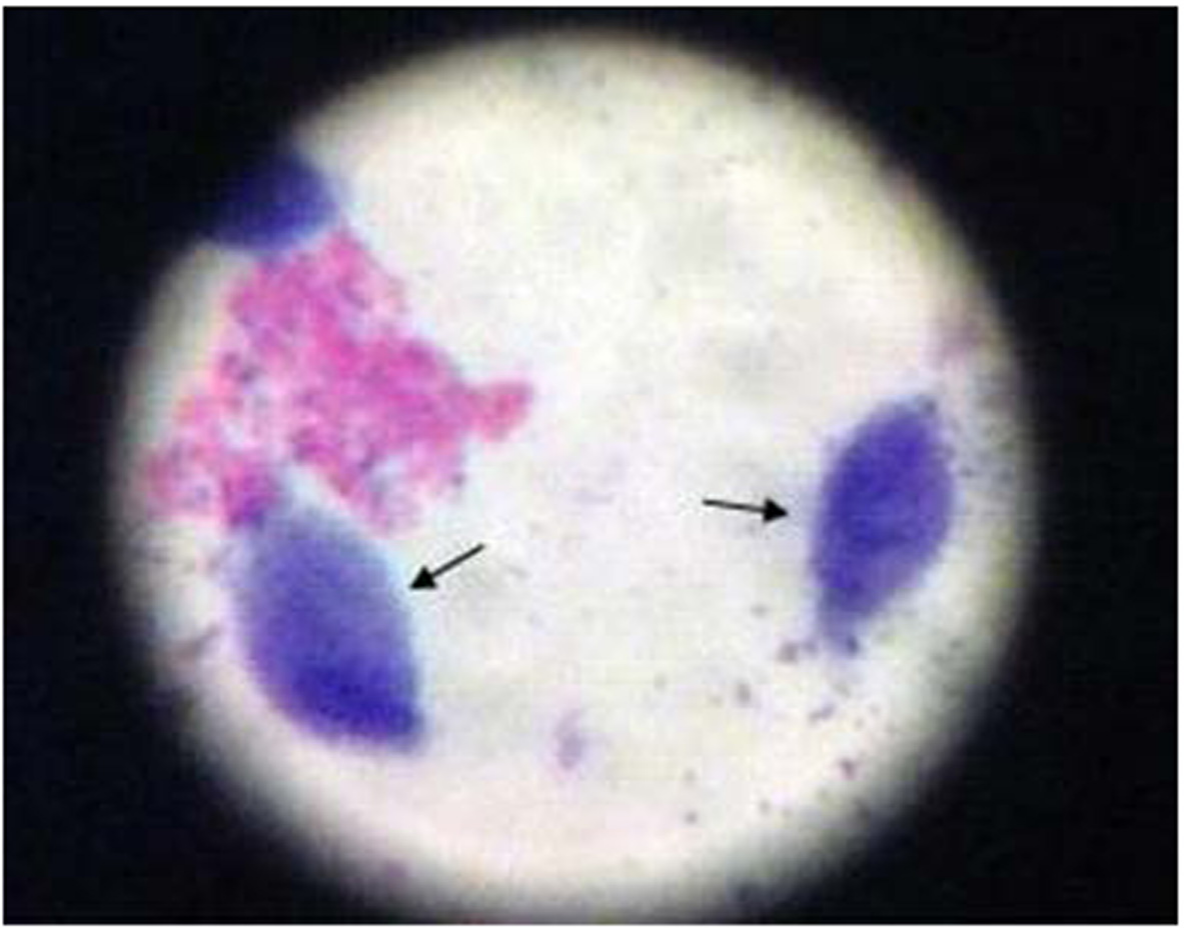
***T. vaginalis*** **after staining with GIEMSA stain × 100.** For Giemsa stain, the vaginal smear was applied to a small area of a clean microscope slide,then allowed to air-dry before fixing it with methanol for about 1 minute and left to dry again. Then, the slide was placed in 20% Giemsa stain for 20 minutes. The slide was then rinsed with buffered water to remove excess stain and then left to dry(no cover slip applied). The stained preparations were examined under the light microscope with oil immersion objective (100 ×) to detect the trophozoites of *Trichomonas vaginalis.*

For Giemsa stain, The vaginal smear was applied on a slide allowed to air-dry before fixing it with methanol for about 1 minute, and then stained with 20% Giemsa to be screened for T. vaginalis trophozoites (Figure 2).

For the Kalon Latex test, the latex is mixed on a slide with theelute from a vaginal swab. Any T. vaginalis antigen present in the sample causes cross-linking (agglutination) of the sensitised latex. After mixing for three minutes, the slide is read. Agglutination of the beads is indicative of T. vaginalis presence.

**Figure 3:**
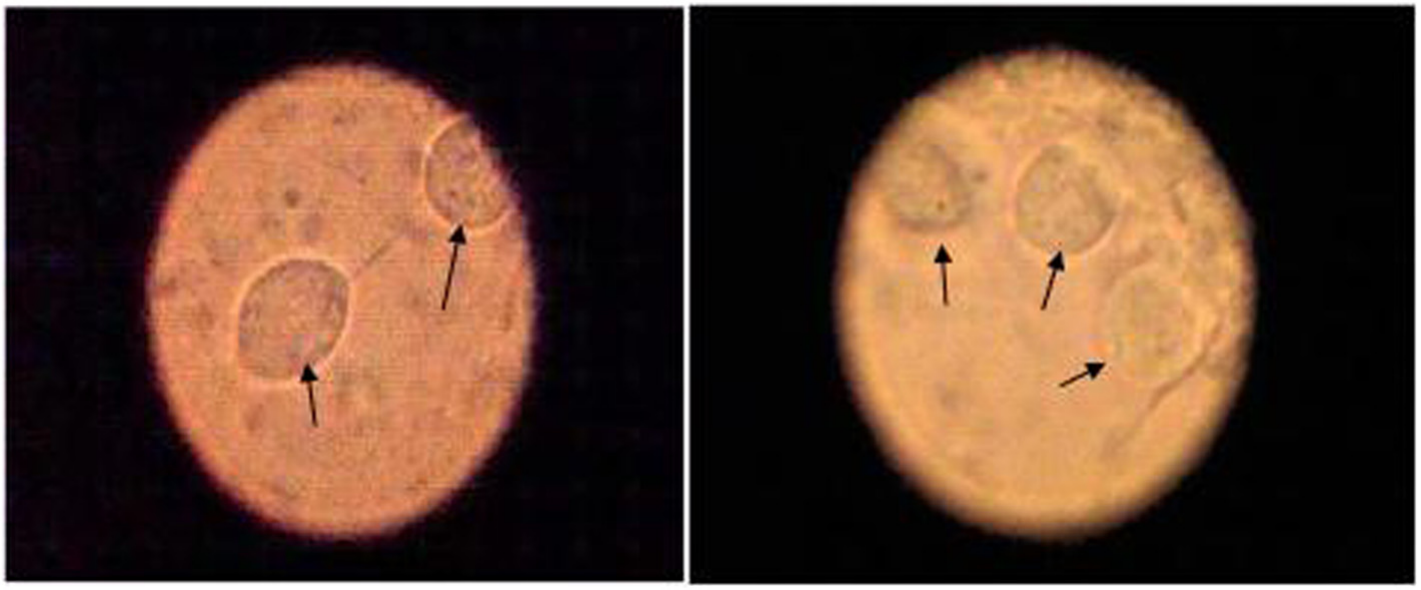
***T. vaginalis*** **in Modified Diamond culture × 40.** Before inoculation, the culture tubes were removed from −20°C and incubated at 37°C for 1 to 2 hours to bring prepared medium at room temperature. Directly, the swab was inserted into the culture tubes containing the modified Diamond's medium. The tubes containing the inoculated medium were incubated vertically at 37°C in 5% CO2. The culture was examined daily using binocular or inverted microscope for the presence of *T. vaginalis* trophozoites. Aseptically, drop of the deposit was removed using a sterile pipette and placed on a slide and covered with a glass cover slip for wet mount examination. Examination was done under I0×-40× magnification. If the organism was not seen, the culture was incubated again for up to 1 week, with daily examination or every other day in the same manner. If no trichomonads were seen, the specimen was considered negative and discarded.

For the Diamond test, the swab was directly inserted into the culture tubes containing, the modified Diamond’s medium, then incubated at 37 degrees in 5% CO2 and examined daily for the presence of T. vaginalis trophozoites (Figure 3). If the organism was not seen, the culture was incubated again for up to 1 week, with daily examination or every other day in the same manner. If no trichomonads were seen, the specimen was considered negative and discarded.

All the patients data, supporting data, check list and excel sheet are available with a specific serial number.

Statistical analysis was performed with SPSS (statistical package for social science) software package (Version 9.0 for Windows). Chi-square test was used to examine the relation between qualitative variables. For quantitative data, comparison between two groups was done using independent sample t-test. Kappa test was used as a measure of agreement between tests. A probability value of p < 0.05 indicated a statistically significant difference.

## Results

1000 patients with symptoms suggestive of T. vaginalis infection were examined using the 4 listed methods in Table 1. The Modified Diamond culture has been considered to be the gold standard test according to many studies [7-9]. So in our study, the M. Diamond test was used as the reference test, to which other tests were compared. Table 2 shows the validity of the other tests in comparison to Diamond test.

**Table 1:** **Positive and negative results of symptomatic women using different test in comparison with the gold standard test (Diamond culture test)**

**Test used**	**M Diamond culture + ve-ve**		Kappa value*	**p value**
	**( n = 30) (n = 970)**			
Kalon test + ve-ve	30 (100%)	20 (2.1%)	0.740	<0.001
	0 (0%)	950 (97.9%)		
Giemsa test + ve-ve	10 (33.3%)	0 (0%)	0.492	<0.001
	20 (66.7%)	970 (100%)		
Wet mount + ve-ve	10 (33.3%)	0 (0%)	0.492	<0.001
	20 (66.7%)	970 (100%)		

*a measure of agreement between the test and modified Diamond culture.

As shown in the table above, 30 symptomatic women were positive for trichomonas infection using the gold standard test. These were also positive using the Kalon Latex test, however the Kalon test detected another 20 cases as positive for trichomonas infection which were however culture negative. However, both Giemsa stain and wet mount detected the same 10 cases that were culture positive accounting for 33.3% of total cases detected by the gold standard reference test. The relation between the results obtained using the gold standard test in comparison to the other tests applied was statistically significant for each test with a strong agreement (kappa = 0.740) for the Kalon test and moderate agreement with culture for the Giemsa stain and wet mount(kappa = 0.492).

**Table 2:** **Validity of different tests compared to M. Diamond culture as a reference method**

**Test**	**Sensitivity**	**Specificity**	**PPV**	**NPV**
Wet mount	33.3%	100%	100%	98%
Giemsa stain	33.3%	100%	100%	98%
Kalon latex test	100%	97.9%	60%	100%

The Kalon Latex test, as shown in the above table, was found to have the highest sensitivity of 100% with no false negatives and with a PPV of 60% and NPV of 100%. The Giemsa stain and the wet mount revealed the highest specificity of 100%, however a low sensitivity of 33.3% with PPV and NPV of 100% and 98% respectively.

Studied individuals were grouped according to their ages into 4 age groups as shown in Table 3, there was no statistical significant difference regarding the T. vaginalis infection distribution in different age groups among females in the childbearing period (p value: 0.627).

**Table 3:** **Relationship of T. vaginalis positive cases and the different age groups**

**Age group (years)**	**Total n of cases**	**n of positive cases (50)**
18- 20	80	10 (12.5%)
21- 30	380	20 (5.3%)
31- 40	390	20 (5.1%)
41- 45	150	0 (0%)
Total	1000	50
P value	0.627	

As shown in the table above , the highest percentage of women with Trichomonas infection were in the age groups 21–30 and 31–40 years, accounting for 80% of total positive cases (40/50). However, no cases were detected after the age of 40 years. This relation between Trichomonas positive cases and different age groups was statistically non-significant (p = 0.627).

Table 4 shows that there is no statistical significant difference in the prevalence of T. vaginalis among females with different symptoms (p = 0.129), among the 50 cases positive for T. vaginalis. Regarding vaginal discharge, 30 patients presented with frothy yellowish discharge with bad odour, 10 patients presented with yellowish discharge, and 10 patients presented with yellowish white discharge .This relation between the nature of the discharge and the diagnosis of trichomonal infection was found to be highly statistically significant (p = 0.0001).

**Table 4:** **Relationship of T. vaginalis positive cases and the different complaints**

**Complain**	**n of cases**	**n of positive cases**
Discharge	750	20 (2.7%)
Discharge and itching	120	10 (8.3%)
Discharge, itching and burning sensation	130	20 (15.4%)
Total	1000	50
P value	0.129	

The above table shows no statistical significant relation between the various complaints and Trichomonas positive cases (p = 0.129).

Out of the total 50 positive cases, 30 cases were using IUD (4.3%), 10 cases were using pills (14.3%) and 10 cases used no contraception (5.3%), which thus revealed a statistically non-significant correlation between the IUD use and trichomonal infection (p value = 0.669).

## Discussion

As we have previously studied other causes of vaginitis in our center, so we limited this study to a large scale cross-sectional study to assess the incidence of Trichomonas.

In the present study, out of 1000 symptomatic female patients examined, 50 were positive for T. vaginalis infection, achieving a prevalence of 5% by all methods done collectively.

This prevalence is in accordance with other studies that showed a prevalence of T. vaginalis infection that ranged from 4.1%- 5.4% [10-12].

However, other studies demonstrated a lower prevalence of T. vaginalis infection ranging from 2.1%- 3.68%. They concluded that, the epidemiology of this infection is changeable and depends on social factors [13-15].

The variations in the prevalence of infection depend on many factors including age, sexual activity, number of sexual partners, other STDs, phase of menstrual cycle, techniques of examination, specimen collection and laboratory technique. Also, may be due to socio-cultural properties of the communities that change from a country to another and from a society to another [16].

The direct wet mount microscopy is a practical, simple, rapid and relatively cheap test but with low sensitivity in diagnosing T. vaginalis infection. Examination should be performed within half an hour of sample collection otherwise the trophozoite characteristic jerky movements will be attenuated or lost by time .It will be difficult to differentiate the parasite from neutrophils or nuclei of vaginal epithelial cells .This method needs an access to a microscope and a well trained physician to identify the parasite. It is still the most common and widely used method for diagnosis of trichomoniasis being a good positive method.

In this study, it was noticed that staining was only useful with heavy T. vaginalis infection because the trophozoites are usually lost from the slide or damaged during processing.

Culture was considered to be the “gold standard” and the reference test to which results of other tests performed in the present study were compared.

According to the investigations done, out of 1000 symptomatic female patients, 50 (5%) were found positive for T. vaginalis infection by all methods done collectively. The Latex agglutination test detected 50 positive cases, 30 of which were also detected by culture. 10 of the culture-positive cases were detected both by staining and by wet mount. Despite that the Modified Diamond culture offered an excellent visualization of trichomonads, yet, it is costly and not available in routine laboratories. In addition, it requires equipment such as incubators. Moreover, it requires daily sampling and microscopic examination from the second to the seventh day for detection of the growth, hence time-consuming with a late diagnosis of infection. Through this period, the infected person continues to spread the infection before receiving therapy.

In our study, sensitivity and specificity were calculated for each method using Modified Diamond culture as the standard method for comparison. The sensitivity of wet mount examination was 33.3%, specificity was 100%, similarly, the sensitivity of Giemsa stain was 33.3% and its specificity was 100%. While, Latex agglutination test was 100% sensitive and its specificity was 97.9%.

Similarly, Darani et al. study found that the sensitivity and specificity of latex test considering culture as golden standard were 100% and 81% respectively; positive and negative predictive values were 60%and 100%, respectively [17]).

The Kalon Latex test used in our study was able to diagnose 50 cases as positive for T. vaginalis, in comparison to the Diamond culture which only gave a positive diagnosis in 30 cases. Whether these apparently false positive cases (20 cases) by the Kalon test are truly false positive or not (i.e. true positive) cannot be resolved unless confirmatory tests- that could be superior to culture are performed along hand -such as PCR. This could be the scope of future studies. So, until then we would have to consider Kalon Latex test as a very sensitive test that is comparable to culture in detection of Trichomonal infection which could be implemented as a good screening test being cheap, easy to perform and with rapid results.

In other words, a positive Kalon test is in itself poor at confirming Trichomonal infection (PPV = 60%) and further investigations must be undertaken; it did, however, correctly identify 100% of all cases (the sensitivity). However as a screening test, a negative result is very good at reassuring that a patient does not have the infection (NPV = 100%) and at this initial screen correctly identifies 97.9% of those who do not have trichomonal infection (the specificity).

## Conclusions

The overall prevalence of Trichomonas infection using all methods of detection collectively was 5% among the studied population of women which could be an overestimated prevalence until superior and sophisticated tests as PCR are performed, to confirm or refute the Kalon test positive but culture negative cases. We recommend large scale a study addressing this issue, since, a lower prevalence of 3% was obtained using modified Diamond’s culture alone as a gold standard test for detection- being superior to all methods used in this study. This could be possibly attributed to the social and cultural conservative nature of the Egyptian society regarding free sexual relations.

Latex agglutination test is a rapid commercially available kit for detection of T. vaginalis antigens as it provides results within 2–3 minutes. It will be an important addition to the techniques available for T. vaginalis detection being more practical as a screening test when there is difficult access to a microscope, when culture media are not available and when rapid diagnostic results are required. Hence, the need for implementing it in STDs screening programs.

We also recommend increasing the health awareness of females in public health units to seek medical advice if there is any excess discharge or any abnormal symptoms. Studies for STI diseases, other than T. vaginalis infection, are recommended to be considered, demonstrating their prevalence and screening methods.
